# Prosthetic Rehabilitation of a Mandibular Defect Using a Malo Bridge: A Case Report

**DOI:** 10.7759/cureus.69298

**Published:** 2024-09-12

**Authors:** Shiney Boruah, Vidyashree V Nandini, Shafath Ahmed, Abinaya Saravanan, Manjula G

**Affiliations:** 1 Prosthodontics, SRM Kattankulathur Dental College and Hospital, Chengalpattu, IND

**Keywords:** implant angulation, malo bridge, mandibular defect, odontogenic keratocysts, prosthetic rehabilitation

## Abstract

Prosthetic rehabilitation is necessary to improve a patient's quality of life after large cysts and tumors are removed. Although odontogenic keratocysts are benign growths, they usually cause extensive localized damage. Large abnormalities on the affected side are often the outcome of surgery. Following an odontogenic keratocyst removal, a large vertical defect was created in the right posterior mandibular region. This article provides a step-by-step description of prosthetic rehabilitation with a Malo bridge. A screw-retained framework with a custom-made abutment is used in Malo bridges, and final crowns are cemented onto it. This concept was applied in this case for the final restoration.

## Introduction

The odontogenic keratocyst (OKC), formerly known as the keratocystic odontogenic tumor (KCOT), is a benign lesion of the jaw with a high recurrence rate and invasive growth. It has a well-defined border with smooth, sclerotic borders on a radiograph [1]. Treatment for OKC is always debatable; it might be aggressive or cautious. Conservative treatment usually entails either curettage or simple enucleation using spoon curettes of marsupialization. On the other hand, aggressive treatment consists of resection, chemical curettage with Carnoy's solution, and peripheral ostectomy [2]. Severe abnormalities in the affected site post-resection may have an adverse effect on the patient's quality of life.

Rehabilitating such problems with prosthetics can be difficult in terms of both function and aesthetics. Prosthetic care of OKC does not offer a straightforward therapeutic option. For the rehabilitation of such a deformity, a prosthesis might be either fixed or removable or a combination with attachments [3]. Nonetheless, since implant insertion with bone graft is a contemporary therapeutic modality that improves patient comfort, appearance, and function, it may be the best course of treatment. There are no clear recommendations for the prosthetic phase in the literature for such cases. Portuguese dentist Dr. Paulo Malo introduced the Paulo Malo bridge, popularly referred to as the Malo bridge, a major advancement in dental implants and prosthetics. Features of a Malo implant bridge include a removable occlusal screw-retained superstructure with customized abutments, a precise adaptation made possible by computer-aided design/manufacturing system fabrication of the framework, and the use of the final crowns cemented to the framework to allay the patient's aesthetic concerns. The All-on-4 concept, which provides a full-arch rehabilitation with just four dental implants, is largely linked to this prosthesis. There is documented proof that Malo bridges are used when implant angulations are unfavorable [4]. This article describes in detail how the prosthetic rehabilitation of a significant defect due to the removal of OKC in the right posterior mandibular region was completed by incorporating a Malo bridge design in a conventional screw-retained prosthesis. To make up for the greater deformity due to soft and hard tissue loss, a screw-retained implant-supported prosthesis with a Paulo Malo bridge design was selected as the prosthetic treatment option.

## Case presentation

**Figure 1:**
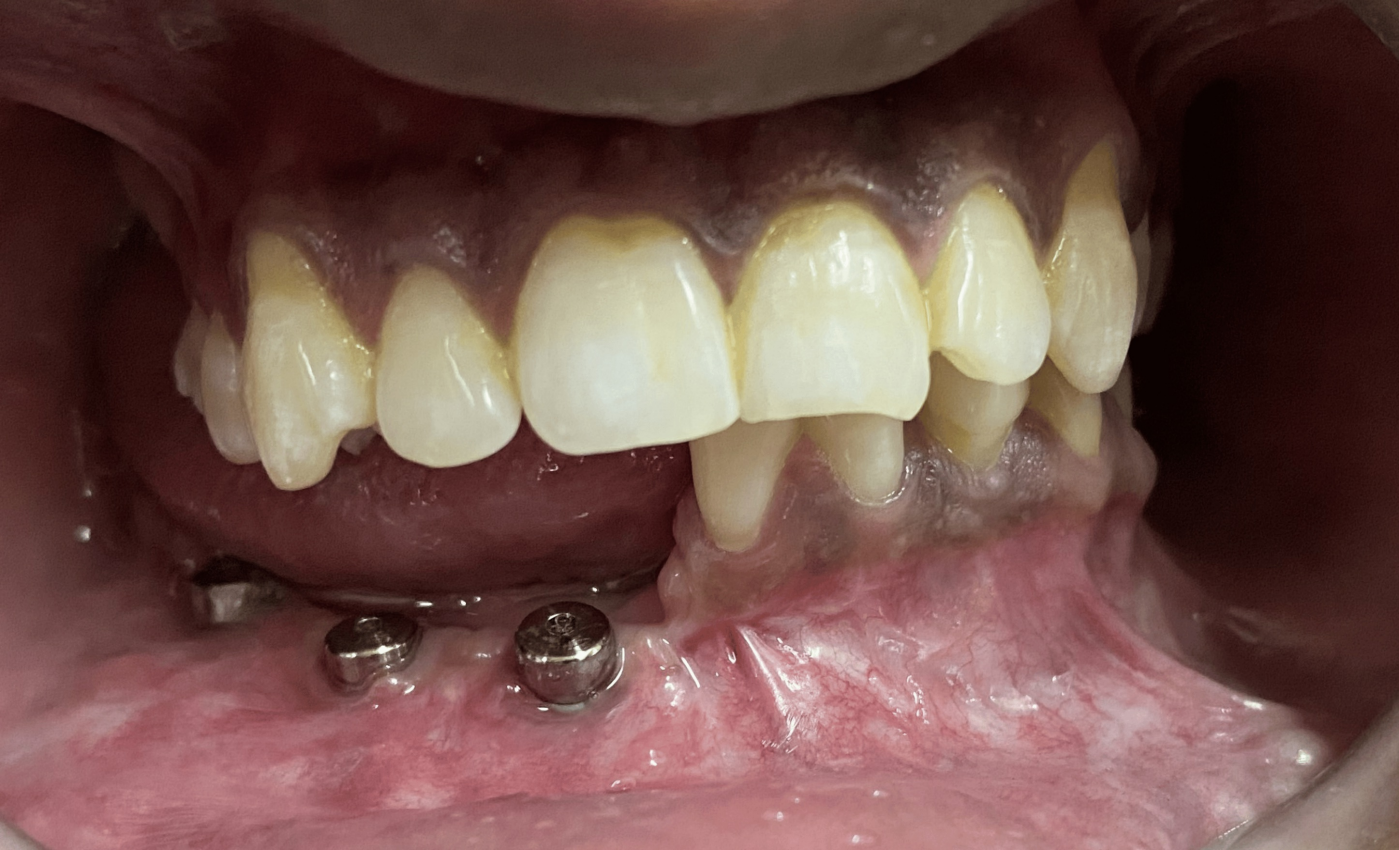
Implants with healing caps on the defect side of the mandible

A 27-year-old male patient reported to the Department of Prosthodontics for the replacement of teeth on the right lower jaw region. The patient discharge summary revealed a swelling on the right lower region extending from the right ear lobe to the lower border of the mandible. There was no history of pain or pus discharge on the site, and there was no evidence of paresthesia. An impacted last molar on the right side of the mandible was present, and the lesion was diagnosed as OKC. Cyst enucleation in the right mandibular region was performed and followed up for a year. Subsequently, bone grafting and immediate implant placement were completed. At this stage, after six months of implant placement, the patient reported for rehabilitation with a prosthesis with special emphasis on function (mastication). An intraoral examination revealed a vertical step deformity on the right lower jaw and missing 41, 42, 43, 44, 45, 46, and 47 teeth. Radiological examination revealed three mandibular endosseous implants, measuring 3.75×11.5 mm, 3.75×13 mm, and 3.5×13 mm (Touareg^TM^-S, Adin Dental Implants, Afula, Israel) placed in the 41, 43, and 45 regions, respectively. The treatment plan was to have an implant-supported prosthesis in the right posterior mandibular area after six months of osseointegration (Figure 1).

Prosthetic rehabilitation

A vertical defect (soft tissue and hard tissue loss) was observed, with compromised implants' parallelism and angulation. The prosthetic challenges included extensive loss of tissue in a vertical direction and implant angulations. The Paulo Malo bridge, a screw-retained implant-supported prosthesis, was planned to be placed in the affected area to compensate for the loss. The proposed restoration included teeth 41 and 42 regions (cemented crowns) and a screw-retained porcelain fused-to-metal fixed prosthesis in teeth 43, 44, and 45 regions with the support of teeth 43 and 45 implants. The prosthesis was designed to restore lost soft and hard tissue, enhancing both appearance and functionality.

Procedure

**Figure 2:**
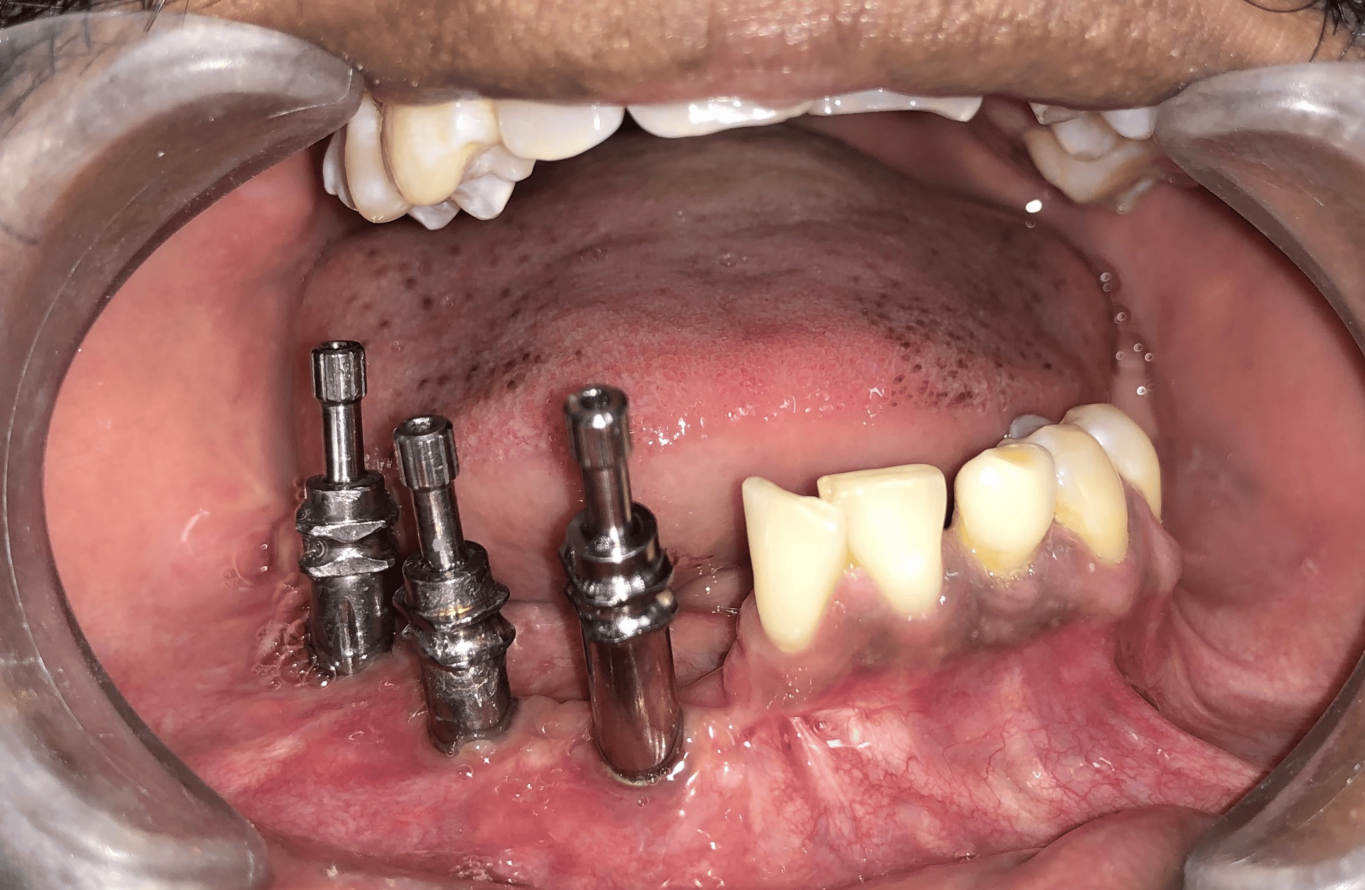
Impression posts attached to the implants for open-tray transfer

An initial impression was made using a stock tray and irreversible hydrocolloid impression material (Chromatex Alginate, DPI, Mumbai, India) three weeks following the placement of the healing abutment (Adin Dental Implants). After the primary cast was obtained, a custom tray was fabricated using auto-polymerizing acrylic resin (DPI RR Cold Cure). The open-tray impression technique was selected as the impression method. The impression posts were splinted using pattern resin (GC pattern resin, PDD, Tokyo, Japan) after they were put in areas 43, 44, and 45 (Adin Implant System), as shown in Figure 2.

**Figure 3:**
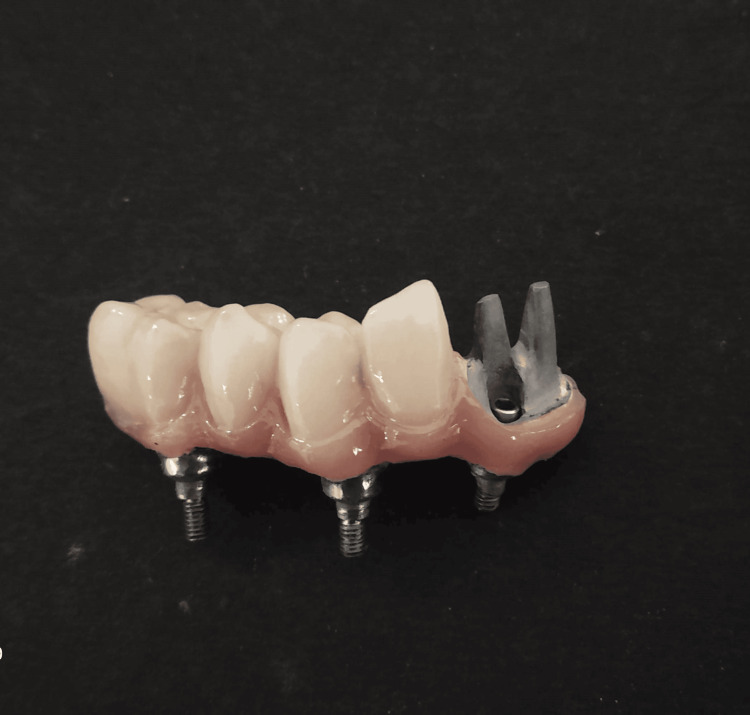
Final screw-retained restoration without the cemented crowns in teeth 41 and 42 regions

An open-tray implant level impression was made using monophase polyvinyl siloxane impression material (Aquasil, Dentsply Sirona, Erlangen, Germany) (Figure 3).

**Figure 4:**
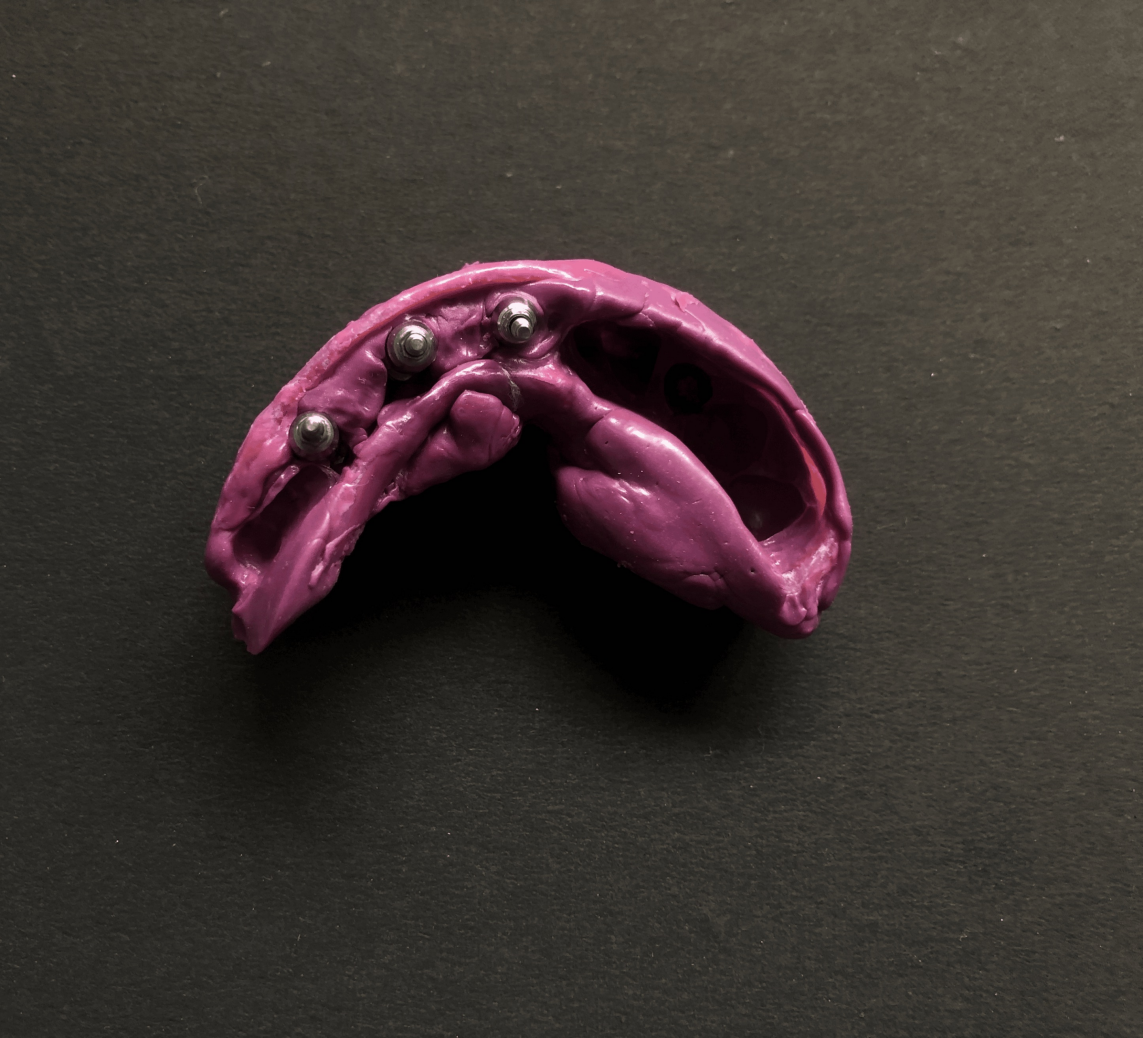
Final impression

**Figure 5:**
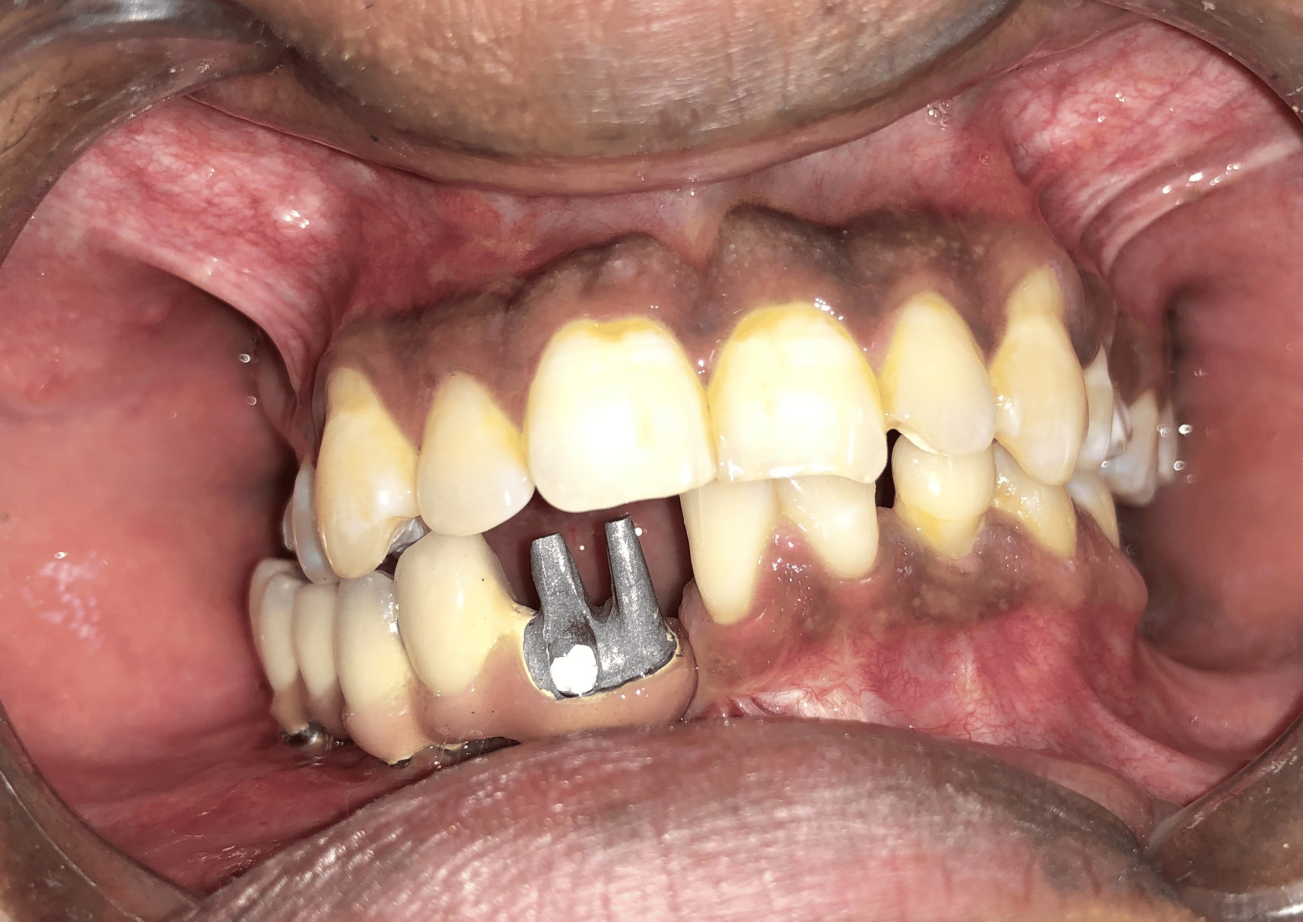
Final restoration in the mouth following the torquing of the abutment screws, without the crowns

**Figure 6:**
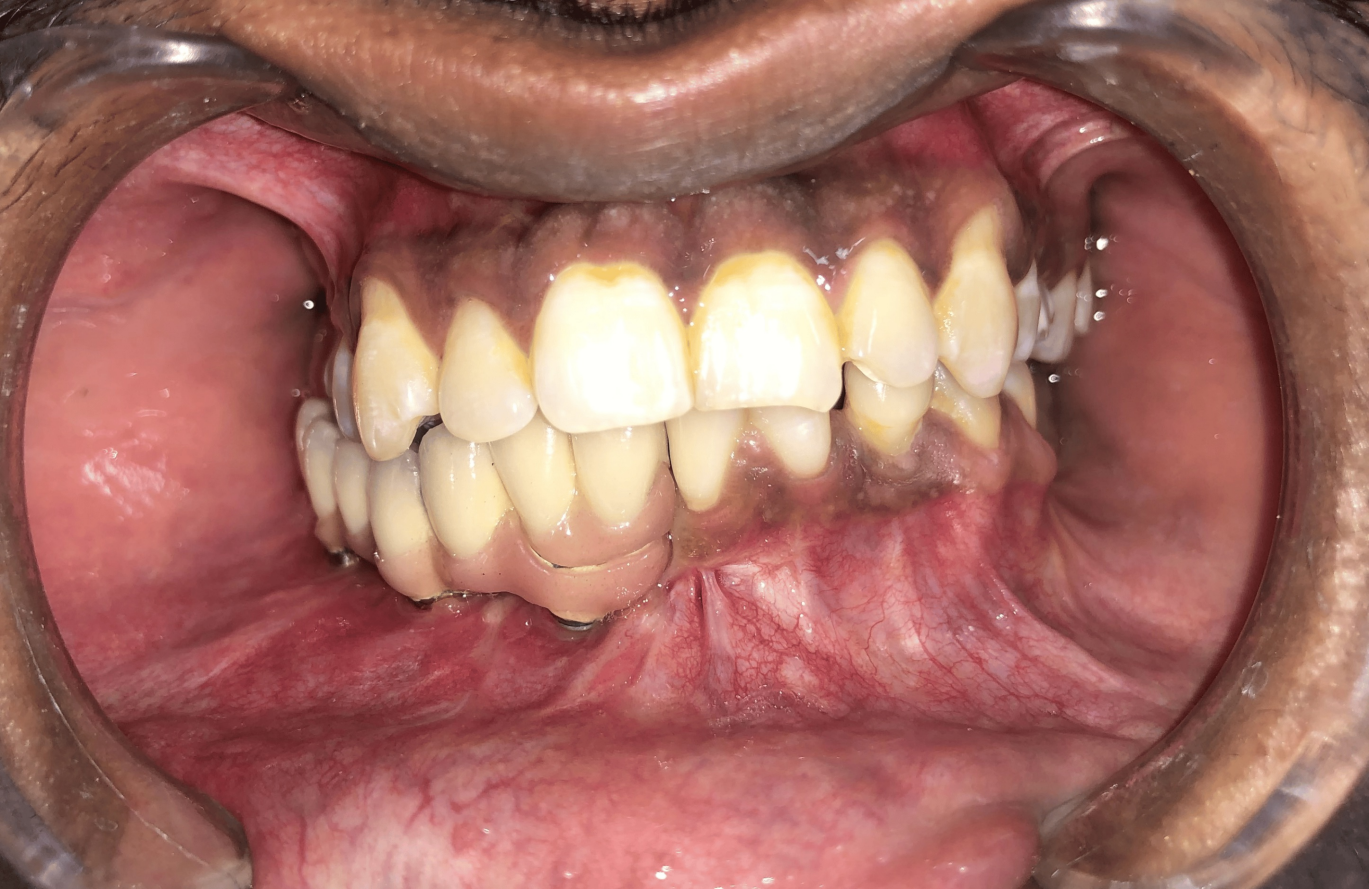
Malo bridge in place following the cementation of teeth 41 and 42

The laboratory counterparts were affixed to the impression posts, transported to the laboratory to create the framework, and intraoral verification was performed. After occlusal correction, the screw-retained implant bridge with Malo prosthesis was inserted by torquing to 15 Ncm (Figures 4-6). The prosthesis was divided into two sections: an anterior piece with two cemented crowns and a posterior section with a screw-retained prosthesis held in place by two implants. The patient was put on a follow-up protocol and given instructions for aftercare. One year after completion of treatment, the patient reported no specific complaints regarding the aesthetics or functionality of the prosthesis.

## Discussion

Rehabilitation of large postsurgical defects with a prosthesis is challenging. Various prosthetic solutions are available, depending on the extent of the afflicted area, including both fixed and removable prostheses. Nonetheless, implant-based prosthetic rehabilitation combined with bone grafting has shown to be an effective means of treating this kind of deformity [5]. In this case report, a significant vertical deficiency on the right posterior mandibular region was treated with prosthetic rehabilitation. To make up for the greater deformity, a screw-retained implant-supported prosthesis with a Malo bridge in the appropriate area was selected as the prosthetic alternative.

In this instance, the prosthetic problems included implants' parallelism and angulation, as they were not prosthetically driven. Rehabilitating an implant that is not aligned correctly is among the most difficult clinical situations that a prosthodontist may face. Based on the clinical condition, the prosthetic solution is individualized, such as telescopic crowns with attachments or fixed/removable prostheses with attachments. There is no report of the use of Malo bridges on a postsurgical OKC. In this instance, a fixed restorative option was considered with the Malo bridge as an option. A screw-retained implant-supported restoration was first fabricated, where the implants were almost parallel. In the anterior region, since the angulation of implants would allow the display of screw access channels on the labial aspect, two cemented crowns were designed to complete the incisor replacement over and above the screw-retained prosthesis. There were no significant complications in this instance other than the orientation of implants and severe soft tissue/hard tissue defects. Prostheses held in place by screws or cement alone are not the best options in such conditions. Predictable retention, retrievability, and the absence of potentially retained subgingival cement are benefits of screw-retained implant restorations.

Nevertheless, there are certain drawbacks, including the requirement for exact implant placement in order to achieve a passive fit and the best possible aesthetic site for the screw access hole. Due to unfavorable implant site and angulation, prosthetic screw holes may be pointed buccally, rendering screw-retained prostheses unsightly. A cement-retained prosthesis was also not desired since it was hard to reach the implant site for cement removal due to the deeper subcrestal depth of the implants [6]. Bozini et al. reported that the most common prosthesis-related problems were acrylic resin veneer fractures and wear of dentures [7]. Therefore, a hybrid prosthesis is also not a desirable alternative in such a clinical scenario.

On the other hand, Sha et al. used a Malo implant bridge to treat individuals who were edentulous and found that it provided greater psychological pleasure and improved masticatory performance than traditional overdentures [8]. Malo bridge with customization of abutment aids a near-precise outcome [9]. Given the aforementioned situation, the Paulo Malo prosthesis, which incorporates the advantages of both techniques, was employed as a therapy option in conjunction with a screw-retained prosthesis. In addition, the Malo prosthesis addresses implant angulation, conceals labial screw holes, restores the arch shape, and improves aesthetics by regaining the pink gingival portion. A one-year follow-up period was allocated to the patient. Success can only be determined by long-term follow-up, which is a limitation in this case.

## Conclusions

The described technique can aid in achieving the greatest possible functional and cosmetic outcomes by carefully considering the ultimate prosthetic goal of function and aesthetics. When considering the restorative phase of implant restorations, it is critical to put the many prosthetic concepts and techniques to use, collaborate with a laboratory that can help ensure a successful outcome, and use these strategies. This method promotes long-term dental health and well-being while also improving patients' quality of life.
